# The receptor binding domain of SARS-CoV-2 spike protein is the result of an ancestral recombination between the bat-CoV RaTG13 and the pangolin-CoV MP789

**DOI:** 10.1186/s13104-020-05242-8

**Published:** 2020-08-27

**Authors:** Alejandro Flores-Alanis, Luisa Sandner-Miranda, Gabriela Delgado, Alejandro Cravioto, Rosario Morales-Espinosa

**Affiliations:** Departamento de Microbiología y Parasitología, Facultad de Medicina, Universidad Nacional Autónoma de México, Mexico City, Mexico

**Keywords:** SARS-CoV-2, Spike glycoprotein, Recombination, Natural selection, Genealogy, Molecular evolution

## Abstract

**Objective:**

In December 2019 a novel coronavirus (SARS-CoV-2) that is causing the current COVID-19 pandemic was identified in Wuhan, China. Many questions have been raised about its origin and adaptation to humans. In the present work we performed a genetic analysis of the Spike glycoprotein (S) of SARS-CoV-2 and other related coronaviruses (CoVs) isolated from different hosts in order to trace the evolutionary history of this protein and the adaptation of SARS-CoV-2 to humans.

**Results:**

Based on the sequence analysis of the S gene, we suggest that the origin of SARS-CoV-2 is the result of recombination events between bat and pangolin CoVs. The hybrid SARS-CoV-2 ancestor jumped to humans and has been maintained by natural selection. Although the S protein of RaTG13 bat CoV has a high nucleotide identity with the S protein of SARS-CoV-2, the phylogenetic tree and the haplotype network suggest a non-direct parental relationship between these CoVs. Moreover, it is likely that the basic function of the receptor-binding domain (RBD) of S protein was acquired by the SARS-CoV-2 from the MP789 pangolin CoV by recombination and it has been highly conserved.

## Introduction

In the last 20 years, six coronaviruses (CoVs) causing respiratory disease in humans have been detected, namely, HCoV-229E, HCoV-OC43, SARS-CoV, HCoV-NL63, HCoV-HKU1, and MERS-CoV, all of which have a zoonotic origin. Genetic analyzes suggest that HCoV-229E, HCoV-NL63, SARS-CoV and MERS-CoV may originate from CoVs found in bats, while HCoV-OC43 and HCoV-HKU1 may have its origin in CoVs found in rodents. Some of these viruses have intermediate hosts between their natural original hosts and humans. HCoV-OC43 could be transmitted by cattle, HCoV-229E and MERS-CoV by camelids, and SARS-CoV by civets [1, 2]. In late 2019, a seventh CoV (SARS-CoV-2) was identified as the cause of Coronavirus Disease 2019 (COVID-19) characterized by fever, cough and dyspnea with more severe disease leading to pneumonia and respiratory distress syndrome [3]. Genomic analyzes suggest that this new virus originated from bats CoVs, specifically from RaTG13 bat CoV, since these CoVs share high level of genomic similarity (96.2%) [4, 5]. However, pangolins have also been suggested as natural reservoirs of SARS-CoV-2 due to theirs genomic similarities that range between 85% and 92% [6–8].

Viruses, like other pathogenic microorganisms, are subject to different evolutionary forces that allow them to adapt and jump from one host to another. Mutation, gene flow and recombination generate genetic variation that is maintained or removed by natural selection and gene drift. One of the key factors in the process of adaptation by CoVs to different species is their ability to infect cells of their new host. CoVs are able to infect cells through a membrane glycoprotein called Spike (S) [1]. This protein contains an amino-terminal subunit 1 (S1) and a carboxyl-terminal subunit 2 (S2) [9]. The S1 has a Receptor-Binding Domain (RBD) that allows the virus to bind to different receptors on cells of its different hosts. For example, MERS-CoV binds to dipeptidyl dipeptidase 4 (DPP4) [10], while SARS-CoV and SARS-CoV-2 bind to the receptor for the Angiotensin-Converting Enzyme 2 (ACE2) [4, 11]. Although these last two viruses bind to the same receptor, their RBDs present variations in the amino acids involved in the ACE2 recognition site [12].

Previous reports show that protein S is one of the most variable regions of the SARS-CoV-2 genome that is under natural selection and where recombination signals have been recorded [5, 7, 13], suggesting that this protein changes constantly and plays an important role in adapting to humans. In our study, we performed a genetic analysis of the protein S of SARS-CoV-2 and related CoVs isolated from different hosts in order to trace the evolutionary history of protein S and the adaptation of SARS-CoV-2 to humans.

## Main text

### Methods

The sequences of the Glycoprotein Spike gene from 76 CoVs isolated from different hosts and 148 clinical isolates of SARS-CoV-2 were retrieved from NCBI’s GenBank [14] and GISAID [15] databases (Additional file 1). The most representative sequence, the China Wuhan H1 sequence, was used as a reference sequence in the figures of this study. The sequences were aligned and analyzed with MAFFT v7.3 [16] and edited using BioEdit v7.2 [17] and Jalview v2.11 [18]. The nucleotide substitution model of the sequence set was determined with the j Modeltest2 program [19] and a phylogenetic tree was obtained using the Mr. Bayes program [20] under a General Time-Reversible substitution plus proportion of invariable sites and rate of variation across sites (GTR + I+ G). We ran 5 Markov Chains Monte Carlo for 500,000 generations and discarded 25% of the initial trees. The consensus tree was edited using the Figtree v1.4.3 program [21]. A haplotype network was built using the PopArt v1.7 program [22]. Nucleotide similarity analysis was carried out using the SimPlot v3.5 program with a 100 bp window at 30 bp step and Kimura 2-parameter model [23]. The DnaSP v5.1 program [24] was used to perform the McDonald-Kreitman (MK) test.

## Results and discussion

**Fig. 1:**
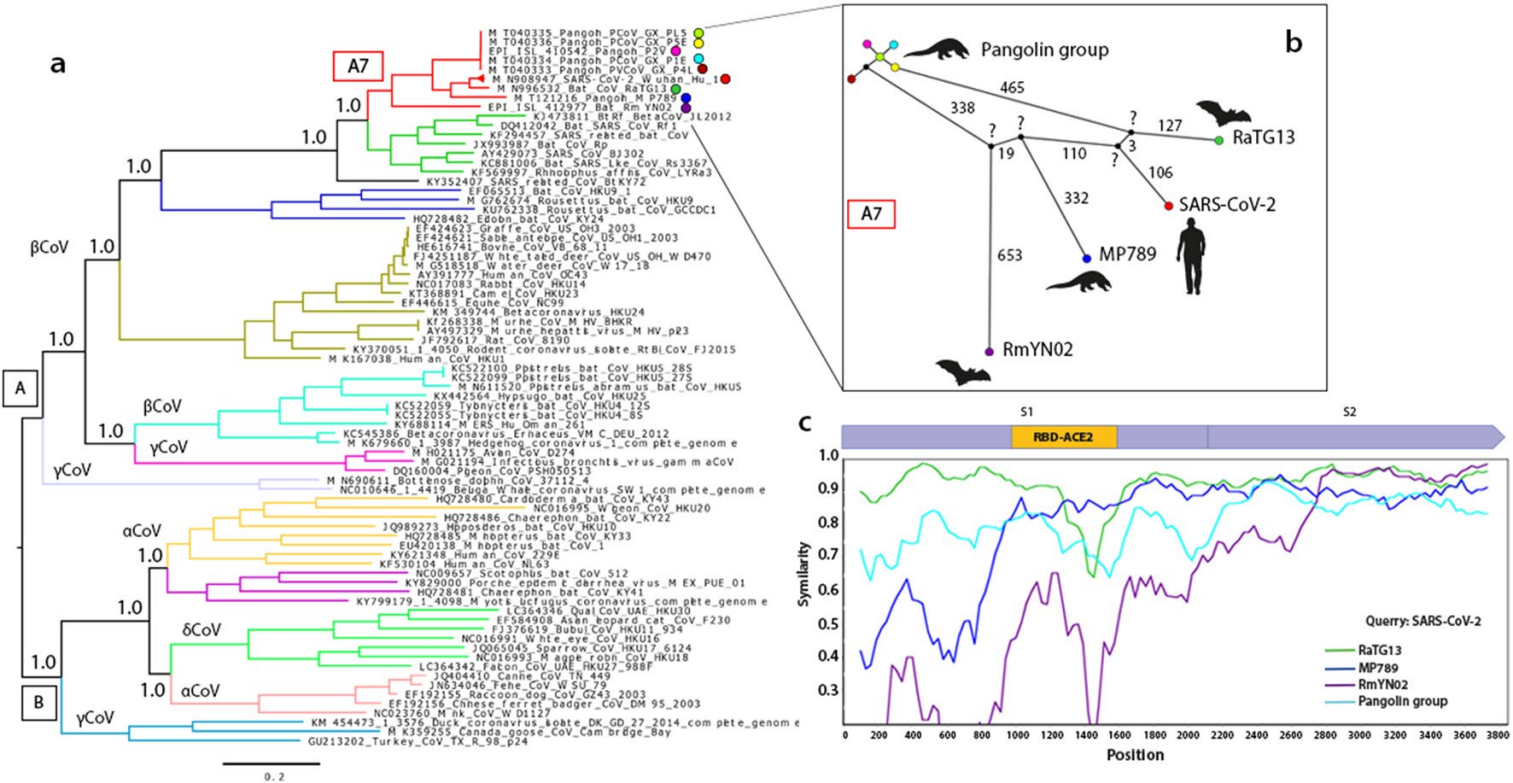
Genetic comparison of S gene of SARS-CoV-2 and other coronaviruses. **a** Phylogenetic tree depicting the relationship between S gene of 76 coronavirus (CoVs) isolates from different hosts and 148 SARS-CoV-2 isolates. CoVs were classified by genus (α, β, γ and δCoVs) and each cluster is shown in a different color. The A7 cluster in red, grouped the SARS-CoV-2 and other related CoVs, each one is marked with a colored circle. The number presented in the nodes indicates posterior probabilities, and the scale bar represents the average number of nucleotide substitutions. All nodes in the A7 cluster had posterior probabilities of 1.0. **b** Haplotype network of S gene, the colored circles represent each CoV according to the A7 cluster in the tree. The PcoV_GX_P5L, P5E, P1E, P4L and P2V were named as Pangolin groups. Small black circles represent hypothetical or unsampled haplotypes. The small black circles, marked with a (?), indicate the hypothetical ancestors of SARS-CoV-2_Wuhan_Hu-1, RaTG13, RaTG13, MP789 and RmYN02. The numbers indicate the number of mutations that separate each haplotype. **c** Patterns of nucleotide sequence identity in the S gene for SARS-CoV-2_Wuhan_Hu-1, RaTG13, MP789, RmYN02 and Pangolin group. Similarity was calculated within sliding window of 300 bp moving with steps of 30 bp

Phylogenetic analysis defined two large clades (A and B). Within clade A, 5 clusters belonging to the betacoronavirus (βCoV) genus and 2 clusters belonging to the gammacoronavirus (γCoV) genus were detected (Fig. 1a). The βCoV cluster A7 corresponds to human SARS-CoV-2 (148 analyzed sequences), 6 pangolin CoVs (*Manis javanica*) (MP789, PcoV_GX_P5L, P5E, P1E, P4L and P2V) and 2 bat CoVs (RaTG13 and RmYN02; *Rhinolophus affinis* and *R. malayanus*, respectively). Within this cluster, we found that SARS-CoV-2 is genetically related to CoV RaTG13, and both share a common ancestor with CoV MP789 (Fig. 1a). This result agrees with previous analyzes made with the complete genome and with S protein [7, 25].

Since we did not find any phylogenetic incongruence reflected on the tree, suggesting a lack of recombination between clusters, we focused more specifically on the cluster where SARS-CoV-2 was found (A7) (Fig. 1a). We analyzed the genealogical relationships between the CoVs that comprise cluster A7. The haplotype network showed the formation of a loop between the group of pangolins and 4 hypothetical ancestral haplotypes (Fig. 1b), suggesting recombination within this cluster. Furthermore, the network suggests that 4 of the isolates analyzed here (SARS-CoV-2, RaTG13, MP789 and RmYN02) diverged from these 4 hypothetical ancestors (Fig. 1b). Despite the fact that SARS-CoV-2 and RaTG13 CoV share a genomic nucleotide identity of 96.2% [4], and an S gene nucleotide identity of 93.15% [7], the divergence showed in the phylogenetic tree and in the haplotype network rules out a direct parental relationship between these two isolates (Fig. 1a and b).

In 2019, various pangolin CoVs were isolated, among which the isolate MP789 CoV is the most interesting because it shares a nucleotide similarity of 85%–92% with SARS-CoV-2, and 90% with RaTG13 CoV [7]. The similarity analysis of the S nucleotide sequences of cluster A7 shows a mosaic similarity pattern across the S gene between SARS-CoV-2, RaTG13, MP789 and RmYN02, which suggests a probable ancestral genetic exchange between the 4 hypothetical ancestors of these CoVs (Fig. 1c). The most notable differences between SARS-CoV-2 and the rest of the CoVs S gene were found in the RBD, indicating a hybrid zone between RaTG13 and MP789 CoVs in this region (Fig. 1c). This result suggests a probable ancestral cross-species recombination between bat and pangolin CoVs.

S protein is thought to be under natural selection and plays an important role in cross-species transmission [5, 26–28]. A recent study reported negative selection in the S gene when SARS-CoV-2 was compared with RaTG13 and a group of pangolin CoVs [26]. We performed an MK test between SARS-CoV-2, RaTG13 and MP789, the results of which showed that between SARS-CoV-2 and RaTG13 CoV there were more synonymous (dS) than nonsynonymous (dN) substitutions, indicating negative selection (NI > 1). Whereas, between SARS-CoV-2 and MP789 CoV the contrary was found, indicating positive selection (NI < 1) (Table 1). The negative selection predicted for SARS-CoV-2 is due to its high similarity to RaTG13 CoV, therefore, the fixation of dN substitutions are not favored. On the other hand, the incongruences found in the pangolin CoV results compared to a previous study [26] could be due to differences in the strategies and methods used.

**Table 1:** McDonald-Kreitman test for Spike gene and RBD of SARS-CoV-2 comparing RaTG13 and MP789 CoVs

S gene	Polymorphic substitutions between virus		MK NI	Fisher’s exact test *p* value
	Nonsynonymous	Synonymous		
RaTG13 CoV	33	223	12.839	0.00000
MP789 CoV	505	75	0.371	0.04134
RBD				
RaTG13 CoV	26	60	ND	0.310345
MP789 CoV	7	71	ND	0.101266

NI Neutrality index (significance at 95%)

ND undetermined

**Fig. 2:**
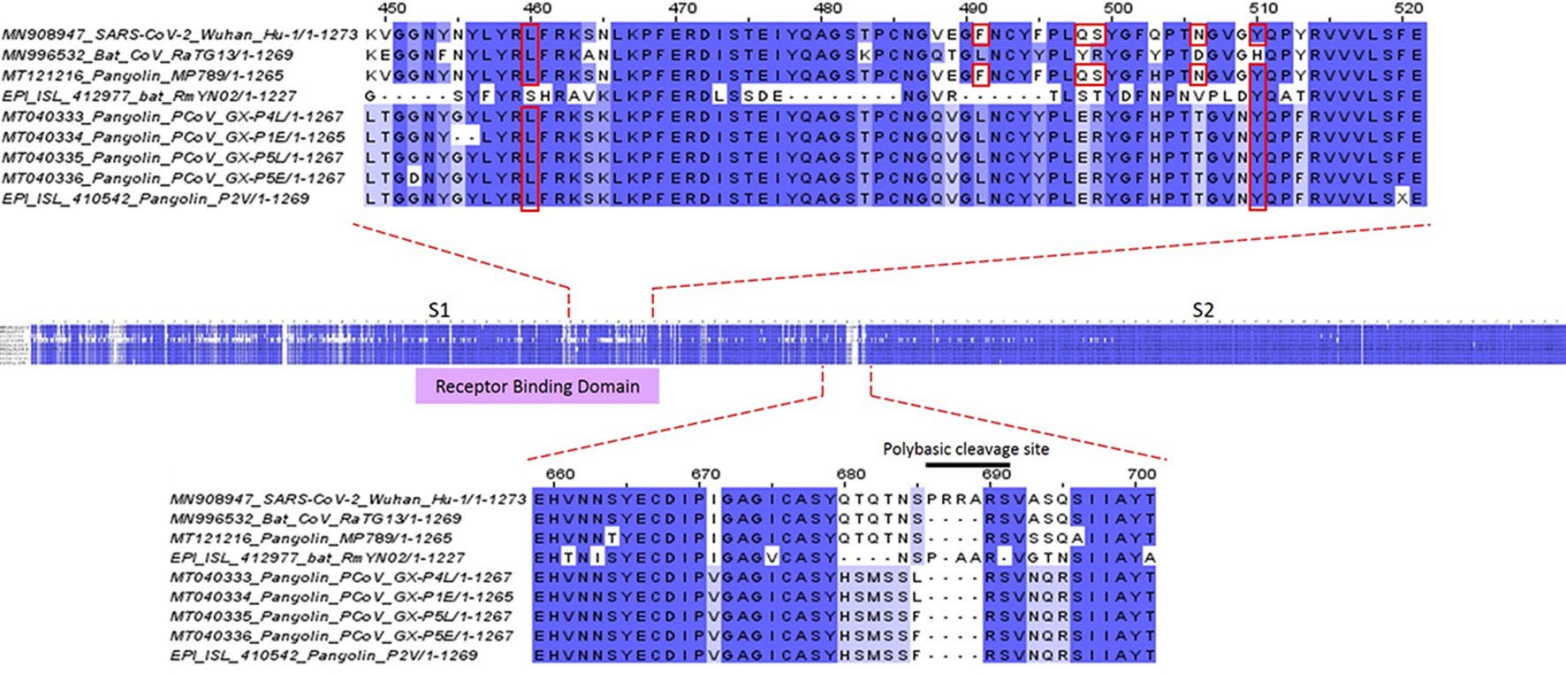
Homology comparison of S protein between coronavirus from different hosts. We compared the SARS-CoV-2_Wuhan_Hu-1 (human), RaTG13 (bat), RmYN02 (bat), MP789 (pangolin) and Pangolin group CoVs (PcoV_GX_P5L, P5E, P1E, P4L and P2V). The residues conserved in all sequences are colored in dark blue, highly conserved residues in medium blue and somewhat conserved residues in light blue. Residues marked with a red box are involved in the contact of the S protein with the ACE2 receptor. Dashes indicate deletions. The polybasic cleavage site is unique in SARS-CoV-2. RmYN02 CoV presents an insertion but this is not a polybasic cleavage site

The S protein RBD plays a key role during the infection process of SARS-CoV-2 to human cells because it contains the six amino acids (L455, F486, Q493, S494, N501 and Y505) that are essential for efficient binding of SARS-CoV-2 to ACE2 [12]. SARS-CoV-2 RBD shows a closer similarity to MP789 CoV RBD (96.8% homology) than to RaTG13 CoV RBD (89.56% homology) [7]. Interestingly, we found that 26 of 33 dN substitutions between SARS-CoV-2 and RaTG13 CoV were located in the RBD, while 7 of 505 dN substitutions between SARS-CoV-2 and MP789 CoV were also located in the RBD (Table 1). This indicates that in this region, MP789 CoV has suffered less dN changes than RaTG13 CoV when compared with SARS-CoV-2. Since only one polymorphism was detected in RBD in the 148 SARS-CoV-2 sequences, the MK test did not determine any value, suggesting that this is a highly conserved region. The comparison between SARS-CoV-2 and MP789 CoV RBD shows that they share the 6 amino acids that are essential for binding to ACE2 receptor, while in RaTG13 CoV these amino acids are missing (Fig. 2). These results could indicate that both the pangolin and humans have similar ACE2 at the interacting domain with S protein, as reported by others [29, 30]. As a consequence, the ACE2 binding sites and the region in general should be conserved (70% homology), being sufficient for the interaction to take place.

A genetic feature that makes SARS-CoV-2 more infectious is the fact that the S protein harbors an insert of 12-nucleotides between the S1 and S2 subunits that encode for a polybasic cleavage site (RRAR) that is recognized by furins (Fig. 2). This cleavage site is related with an increased efficiency of entry during infection [31, 32]. Nevertheless, this insertion is not present in all betacoronaviruses, like in SARS-CoV [13, 31, 33]. However, the human HKU1 CoV and MERS-CoV have variants of polybasic insertions that are also recognized by furins [34–36]. The presence of these polybasic insertions have been seen to increase the pathogenicity of viruses, such as in avian influenza [37–39], MERS-like CoV [40], and in bovine CoV [41].

We also found that RmYN02 CoV has an insert in the same position as that in SARS-CoV-2, but it is not a polybasic cleavage insertion (-AAR). There have been suggestions that instead, it could be the product of recombination between wild bat CoVs [13]. On the other hand, several experiments have shown that this polybasic cleavage site is acquired and fixed during the serial passage of CoVs in cell cultures or in animals [37, 42]. The aforementioned leads to two possible explanations for the polybasic cleavage insertion in SARS-CoV-2 and the role in its adaptation to humans: (1) the ancestor of SARS-CoV-2 acquired it in a host, went through a recombination process in an unidentified intermediary host, and then jumped to humans, or (2) it was acquired in humans during a cycle of human to human transmission that helped its adaptation and virulence process. Rambaut et al. [43] determined that the most recent common ancestor of SARS-CoV-2 appeared in November 2019 and proposes that the virus had enough time to acquire the insert during transmission between humans.

## Conclusion

Whether the ancestral CoV that gave rise to SARS-CoV-2 came from a bat or a pangolin is still not yet known, but based on the analysis of the S gene performed in this study, we suggest that it is more likely to have come from a bat. However, the region essential for human ACE2 (RBD) binding is a hybrid between RaTG13 and MP789 CoVs, and it is likely that the basic function of the RBD was acquired by the SARS-CoV-2 from the MP789 CoV by recombination with an ancestral CoV, which had a RaTG13 genomic background. Subsequently, the SARS-CoV-2 ancestor jumped to humans where the S protein has been maintained by positive selection and where the RBD has been highly conserved. This illustrates the complexity of CoV cross-species infection dynamics and the relevance of genetic exchange between CoVs. In the future, molecular epidemiological surveillance studies in wild isolates will be vital for identifying genetic changes in viruses that could result in novel adaptations to humans and thereby, enabling the development of another pandemic, such as the one we are currently experiencing.

## Limitations

Here we mentioned two characteristics in S protein as key factors in the adaptation and infectivity of SARS-CoV-2, the presence of six amino acids essentials for binding to ACE2 receptor and the polybasic cleavage insert. Nevertheless, the knowledge about this virus has increased very fast and new possible features involved in virus adaptation to human beings has been reported. To fully understand the origin and adaptation of this virus, it would be necessary to encourage the extensive sampling of wild animals associated with coronaviruses and perform deeper and wider genomic analyses.

## Supplementary information

**Additional file 1: Table S1.** Information of the sequences of SARS-Cov-2 and other CoVs used in this study. Accession number, isolate, origin, date of collection, host, host common name, sequence size and data source of the Spike gene sequences used in this study.

## Data Availability

The datasets used and/or analyzed during the current study are available in the Additional file 1.

## Abbreviations

SARS-CoV-2: Severe Acute Respiratory Sindrome Coronavirus 2
COVID-19: Coronavirus Disease 2019
CoV: Coronavirus
S: Spike
RBD: Receptor-binding domain
S1: Spike protein sub-unit 1
S2: Spike protein sub-unit 2
ACE2: Angiotensin-Converting Enzyme 2
DPP4: Dipeptidyl dipeptidase 4
NCBI: National Center for Biotechnology Information
GISAID: Global Initiative on Sharing All Influenza Data
bp: Base pairs
βCoV: Betacoronavirus
γCoV: Gammacoronavirus
dS: Synonymous substitutions
dN: Nonsynonymous substitutions
NI: Neutrality index

## References

[1] Ye ZW, Yuan S, Yuen KS, Fung SY, Chan CP, Jin DY (2020). Zoonotic origins of human coronaviruses. Int J Biol Sci.

[2] Cui J, Li F, Shi ZL (2019). Origin and evolution of pathogenic coronaviruses. Nat Rev Microbiol.

[3] Huang C, Wang Y, Li X, Ren L, Zhao J, Hu Y, Zhang L, Fan G, Xu J, Gu X, Cheng Z, Yu T, Xia J, Wei Y, Wu W, Xie X, Yin W, Li H, Liu M, Xiao Y, Gao H, Guo L, Xie J, Wang G, Jiang R, Gao Z, Jin Q, Wang J, Cao B (2020). Clinical features of patients infected with 2019 novel coronavirus in Wuhan, China. Lancet.

[4] Zhou P, Lou YX, Wang XG, Hu B, Zhang L, Zhang W, Si HR, Zhu Y, Li B, Huang CL, Chen HD, Chen J, Luo Y, Guo H, Di Jiang R, Liu MQ, Chen Y, Shen XR, Wang X, Zheng XS, Zhao K, Chen QJ, Deng F, Liu LL, Yan B, Zhan FX, Wang YY, Xiao GF, Shi ZL (2020). A pneumonia outbreak associated with a new coronavirus of probable bat origin. Nature.

[5] Andersen KG, Rambaut A, Lipkin WI, Holmes EC, Garry RF (2020). The proximal origin of SARS-CoV-2. Nat Med.

[6] Lam TTY, Shum MHH, Zhu HC, Tong YG, Ni XB, Liao YS, Wei W, Cheung WYM, Li WJ, Li LF, Leung GM, Holmes EC, Hu YL, Guan Y (2020). Identifying SARS-CoV-2 related coronaviruses in Malayan pangolins. Nature.

[7] Liu P, Jiang J-Z, Wan X-F, Hua Y, Li L, Zhou J, Wang X, Hou F, Chen J, Zou J, Chen J (2020). Are pangolins the intermediate host of the 2019 novel coronavirus (SARS-CoV-2)?. PLoS Pathog.

[8] Zhang T, Wu Q, Zhang Z (2020). Probable Pangolin Origin of SARS-CoV-2 Associated with the COVID-19 Outbreak. Curr Biol.

[9] Wrapp D, Wang N, Corbett KS, Goldsmith JA, Hsieh CL, Abiona O, Graham BS, McLellan JS (2020). Cryo-EM structure of the 2019-nCoV spike in the prefusion conformation. Science.

[10] Reusken CBEM, Raj VS, Koopmans MP, Haagmans BL (2016). Cross host transmission in the emergence of MERS coronavirus. Curr Opin Virol.

[11] Li F, Li W, Farzan M, Harrison SC (2005). Structural biology: Structure of SARS coronavirus spike receptor-binding domain complexed with receptor. Science.

[12] Wan Y, Shang J, Graham R, Baric RS, Li F (2020). Receptor recognition by the novel coronavirus from Wuhan: an analysis based on decade-long structural studies of SARS coronavirus. J Virol.

[13] Zhou H, Chen X, Hu T, Li J, Song H, Liu Y, Wang P, Liu D, Yang J, Holmes EC, Hughes AC, Bi Y, Shi W (2020). A novel bat coronavirus closely related to SARS-CoV-2 contains natural insertions at the S1/S2 cleavage site of the spike protein. Curr Biol..

[14] NCBI SARS-CoV-2 Resources—NCBI. (https://www.ncbi.nlm.nih.gov/sars-cov-2/).

[15] Elbe S, Buckland-Merrett G (2017). Data, disease and diplomacy: GISAID’s innovative contribution to global health. Glob Challenges.

[16] Katoh K, Toh H (2010). Parallelization of the MAFFT multiple sequence alignment program. Bioinformatics.

[17] Hall TA (1999). BioEdit: a user-friendly biological sequences alignment editor and analysis program for Windows 95/98/NT. Nucleic Acids Symp Ser.

[18] Waterhouse AM, Procter JB, Martin DMA, Clamp M, Barton GJ (2009). Sequence analysis Jalview Version 2-a multiple sequence alignment editor and analysis workbench. Bioinforma Appl NOTE.

[19] Darriba D, Taboada GL, Doallo R, Posada D (2012). JModelTest 2: more models, new heuristics and parallel computing. Nat Method..

[20] Ronquist F, Teslenko M, Van Der Mark P, Ayres DL, Darling A, Ohna SH¨, Larget B, Liu L, Suchard MA, Huelsenbeck JP (2012). Software for systematics and evolution MrBayes 3.2: efficient bayesian phylogenetic inference and model choice across a large model space. Syst Biol.

[21] FigTree. (http://tree.bio.ed.ac.uk/software/figtree/).

[22] Clement M, Posada D, Crandall KA (2000). TCS: a computer program to estimate gene genealogies. Mol Ecol.

[23] Lole KS, Bollinger RC, Paranjape RS, Gadkari D, Kulkarni SS, Novak NG, Ingersoll R, Sheppard HW, Ray SC (1999). Full-length human immunodeficiency virus type 1 genomes from subtype c-infected seroconverters in India, with evidence of intersubtype recombination. J Virol.

[24] Librado P, Rozas J (2009). DnaSP v5: a software for comprehensive analysis of DNA polymorphism data. Bioinforma Appl NOTE.

[25] Tang X, Wu C, Li X, Song Y, Yao X, Wu X, Duan Y, Zhang H, Wang Y, Qian Z, Cui J, Lu J (2020). On the origin and continuing evolution of SARS-CoV-2. Natl Sci Rev.

[26] Li X, Giorgi EE, Marichannegowda MH, Foley B, Xiao C, Kong X-P, Chen Y, Gnanakaran S, Korber B, Gao F (2020). Emergence of SARS-CoV-2 through recombination and strong purifying selection. Sci Adv.

[27] Graham RL, Baric RS (2010). Recombination, reservoirs, and the modular spike: mechanisms of coronavirus cross-species transmission. J Virol.

[28] Cagliani R, Forni D, Clerici M, Sironi M (2020). Computational inference of selection underlying the evolution of the novel coronavirus, severe acute respiratory syndrome coronavirus 2. J Virol.

[29] Liu Z, Xiao X, Wei X, Li J, Yang J, Tan H, Zhu J, Zhang Q, Wu J, Liu L (2020). Composition and divergence of coronavirus spike proteins and host ACE2 receptors predict potential intermediate hosts of SARS-CoV-2. J Med Virol.

[30] Zhao X, Chen D, Szabla R, Zheng M, Li G, Du P, Zheng S, Li X, Song C, Li R, Guo J-T, Junop M, Zeng H, Lin H: Broad and differential animal ACE2 receptor usage by SARS-CoV-2. J Virol. 2020. JVI.00940-20. 10.1128/JVI.00940-20. 10.1128/JVI.00940-20 PMC7459545 32661139

[31] Hoffmann M, Kleine-Weber H, Pöhlmann S (2020). A multibasic cleavage site in the spike protein of SARS-CoV-2 is essential for infection of human lung cells. Mol Cell.

[32] Wang Q, Qiu Y, Li JY, Zhou ZJ, Liao CH, Ge XY (2020). A unique protease cleavage site predicted in the spike protein of the novel pneumonia coronavirus (2019-nCoV) potentially related to viral transmissibility. Virologica Sinica.

[33] Zhao X, Chen D, Szabla R, Zheng M, Li G, Du P, Zheng S, Li X, Song C, Li R, Guo J-T, Junop M, Zeng H, Lin H: Broad and differential animal ACE2 receptor usage by SARS-CoV-2. bioRxiv 2020:2020.04.19.048710. 10.1128/JVI.00940-20 PMC7459545 32661139

[34] Coutard B, Valle C, de Lamballerie X, Canard B, Seidah NG, Decroly E (2020). The spike glycoprotein of the new coronavirus 2019-nCoV contains a furin-like cleavage site absent in CoV of the same clade. Antiviral Res.

[35] Chan C-M, Woo PCY, Lau SKP, Tse H, Chen H-L, Li F, Zheng B-J, Chen L, Huang J-D, Yuen K-Y (2008). Spike protein, S, of human coronavirus HKU1: role in viral life cycle and application in antibody detection. Exp Biol Med.

[36] Park JE, Li K, Barlan A, Fehr AR, Perlman S, McCray PB, Gallagher T (2016). Proteolytic processing of middle east respiratory syndrome coronavirus spikes expands virus tropism. Proc Natl Acad Sci U S A.

[37] Ito T, Goto H, Yamamoto E, Tanaka H, Takeuchi M, Kuwayama M, Kawaoka Y, Otsuki K (2001). Generation of a highly pathogenic avian influenza a virus from an avirulent field isolate by passaging in chickens. J Virol.

[38] Monne I, Fusaro A, Nelson MI, Bonfanti L, Mulatti P, Hughes J, Murcia PR, Schivo A, Valastro V, Moreno A, Holmes EC, Cattoli G (2014). Emergence of a highly pathogenic avian influenza virus from a low-pathogenic progenitor. J Virol.

[39] Nao N, Yamagishi J, Miyamoto H, Igarashi M, Manzoor R, Ohnuma A, Tsuda Y, Furuyama W, Shigeno A, Kajihara M, Kishida N, Yoshida R, Takadaa A (2017). Genetic predisposition to acquire a polybasic cleavage site for highly pathogenic avian influenza virus hemagglutinin. MBio..

[40] Menachery VD, Dinnon KH, Yount BL, McAnarney ET, Gralinski LE, Hale A, Graham RL, Scobey T, Anthony SJ, Wang L, Graham B, Randell SH, Baric RS (2019). Lipkin WI.

[41] Chouljenko VN, Lin XQ, Storz J, Kousoulas KG, Gorbalenya AE (2001). Comparison of genomic and predicted amino acid sequences of respiratory and enteric bovine coronaviruses isolated from the same animal with fatal shipping pneumonia. J Gen Virol.

[42] Borucki MK, Allen JE, Chen-Harris H, Zemla A, Vanier G, Mabery S, Torres C, Hullinger P, Slezak T (2013). The role of viral population diversity in adaptation of bovine coronavirus to new host environments. PLoS One.

[43] Phylodynamic Analysis| 176 genomes| 6 Mar 2020-Novel 2019 coronavirus/nCoV-2019 genomic epidemiology—virological. (https://virological.org/t/phylodynamic-analysis-176-genomes-6-mar-2020/356).

